# P-2139. Association Between Respiratory Viral Detection, Mortality, and Postoperative Outcomes in Pediatric Liver Transplant Recipients

**DOI:** 10.1093/ofid/ofaf695.2302

**Published:** 2026-01-11

**Authors:** Juan S Calderon Cardenas, Juan D Bustos Sanchez, Martha I Álvarez-Olmos, Jairo Rivera, Natalia Lucena, Maira Ureña, Maria Alejandra Prieto, Jaime Fernández-Sarmiento

**Affiliations:** Fundación Cardioinfantil - LaCardio, Bogota, Distrito Capital de Bogota, Colombia; Fundación Cardioinfantil - LaCardio, Bogota, Distrito Capital de Bogota, Colombia; Fundación Cardioinfantil - LaCardio, Bogota, Distrito Capital de Bogota, Colombia; Fundación Cardioinfantil - LaCardio, Bogota, Distrito Capital de Bogota, Colombia; Fundación Cardioinfantil - LaCardio, Bogota, Distrito Capital de Bogota, Colombia; Fundación Cardioinfantil - LaCardio, Bogota, Distrito Capital de Bogota, Colombia; Fundación Cardioinfantil - LaCardio, Bogota, Distrito Capital de Bogota, Colombia; Fundación Cardioinfantil - LaCardio, Bogota, Distrito Capital de Bogota, Colombia

## Abstract

**Background:**

Children undergoing liver transplantation are at risk for perioperative complications, and respiratory viral detection during this period may influence post-surgical outcomes. The impact of viral infections on mortality and complications in pediatric liver transplant recipients remains unclear.

**Figure:**
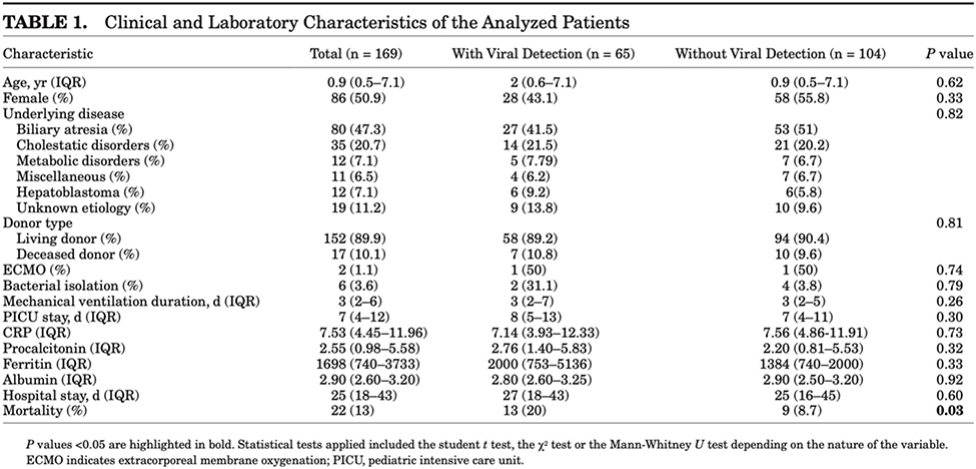

**Figure:**
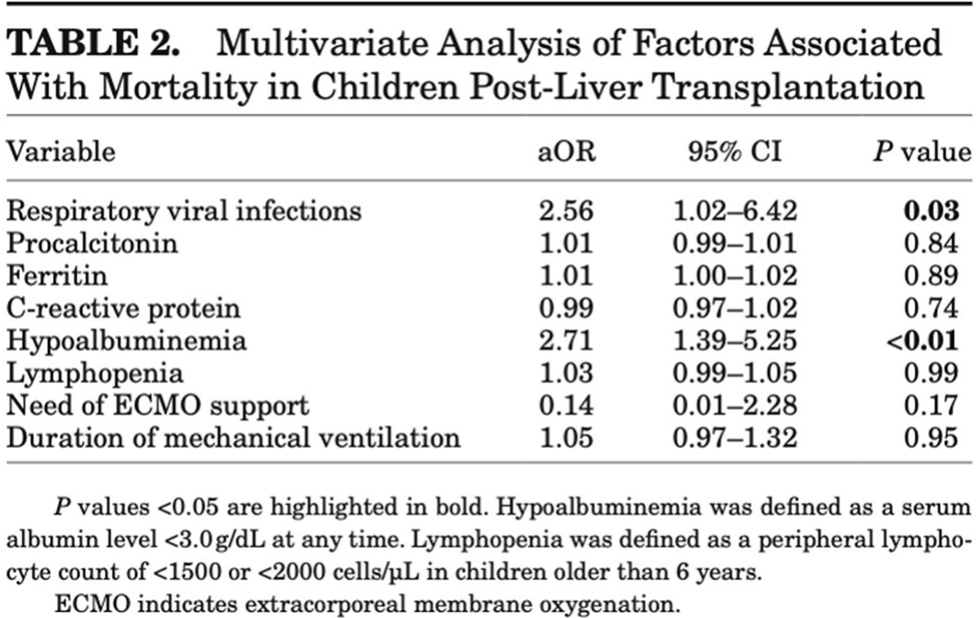

**Methods:**

We conducted a retrospective cohort study (January 2020 - December 2023) at a tertiary university hospital in Bogotá, Colombia. Children undergoing liver transplantation were tested for respiratory viruses via multiplex PCR within 7 days before and 14 days after transplantation. The primary outcome was 28-day post-transplant mortality, while secondary outcomes included postoperative complications, length of hospital stay, and mechanical ventilation duration.

**Figure:**
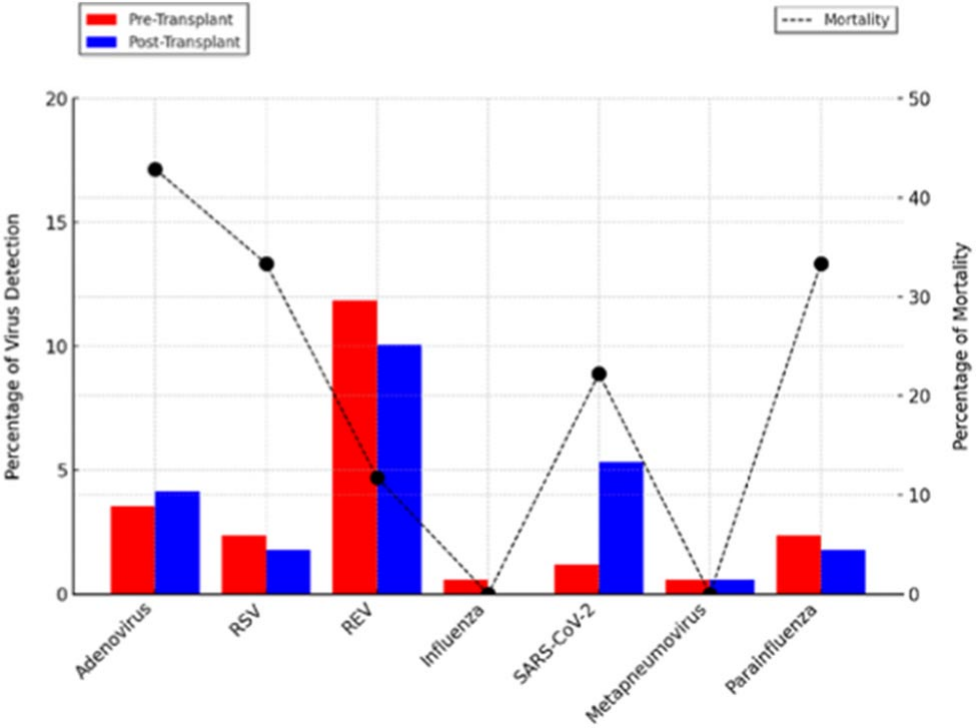

**Figure:**
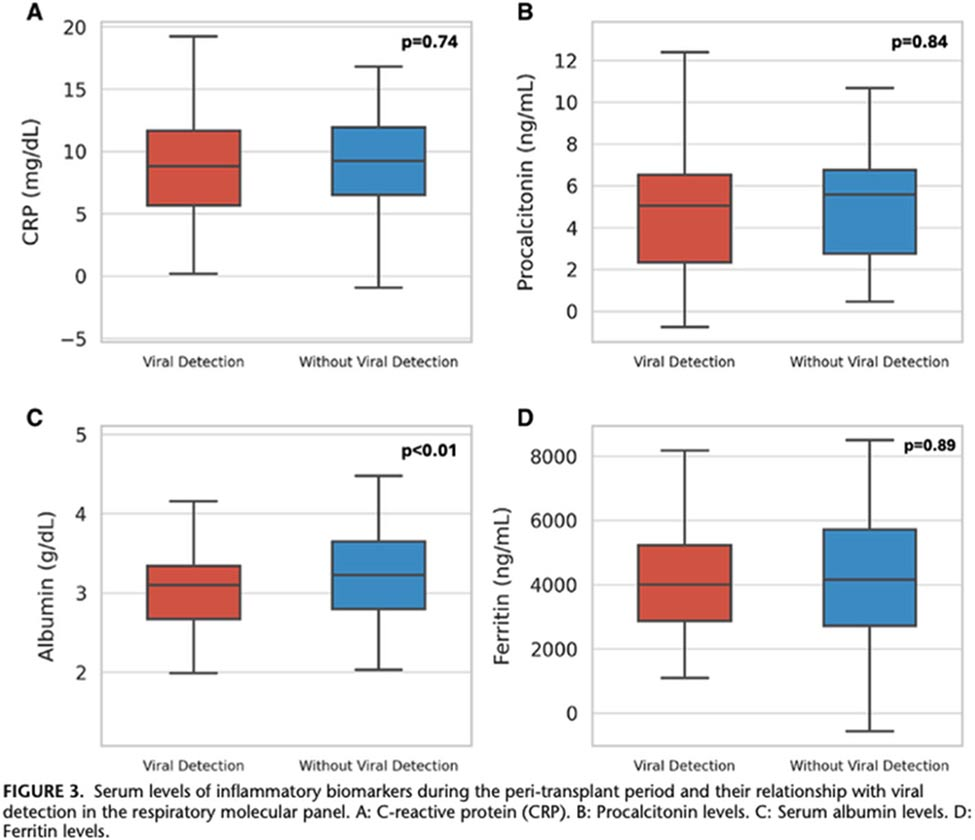

**Results:**

A total of 169 children (median age 0.9 years) underwent liver transplantation. Respiratory viral detection occurred in 38.5% (65/169), with rhinovirus/enterovirus, adenovirus, and parainfluenza being the most common. Mortality was higher in children with viral detection (20% vs. 8.7%, aOR 2.56, 95% CI 1.02-6.42, P=0.03). Bacterial co-infection further increased mortality risk (aOR 2.64, 95% CI 1.06-6.61, P=0.03). Viral detection was also associated with increased surgical complications (aOR 2.18, 95% CI 1.12-4.27, P=0.02). No significant differences were observed in mechanical ventilation duration or hospital length of stay.

**Conclusion:**

Respiratory viral detection in the peri-liver transplant period was associated with increased mortality and surgical complications in pediatric recipients. These findings highlight the need for preoperative viral screening and risk stratification to optimize post-transplant outcomes.

**Disclosures:**

All Authors: No reported disclosures

